# Identification of hepatocellular carcinoma-related genes associated with macrophage differentiation based on bioinformatics analyses

**DOI:** 10.1080/21655979.2020.1868119

**Published:** 2021-01-18

**Authors:** Jun Cao, Chi Zhang, Guo-Qing Jiang, Sheng-Jie Jin, Qian Wang, Ao-Qing Wang, Dou-Sheng Bai

**Affiliations:** Department of Hepatobiliary Surgery, Clinical Medical College, Yangzhou University, Yangzhou, Jiangsu, P.R. China

**Keywords:** Hepatocellular carcinoma, macrophage differentiation, bioinformatic analysis, tumor immune

## Abstract

Macrophage differentiation is associated with tumorigenesis, including the tumorigenesis of hepatocellular carcinoma (HCC). Herein, we explored the value of macrophage differentiation-associated genes (MDGs) in the prognosis of HCC using data from The Cancer Genome Atlas (TCGA) and the International Cancer Genome Consortium (ICGC) databases. We performed multivariate Cox regression analyses to identify the hub genes affecting HCC patient prognoses. The correlations between hub genes and macrophage differentiation and immune checkpoint inhibitors (PD-1, PD-L1, and CTLA4) were investigated. Finally, the potential mechanism was examined with gene set enrichment analysis (GSEA). In total, seventeen differentially expressed MDGs were obtained after intersecting data from the two databases. Multivariate analysis indicated that CDC42 expression was an independent prognostic indicator in both databases. Furthermore, CDC42 showed a strong correlation with the tumor infiltration levels of immune cells in HCC tissue. Correlation analysis revealed that CDC42 expression was positively associated with M2 macrophage markers and immune checkpoint inhibitors, which indicated that CDC42 expression might be related to M2 macrophage differentiation and HCC cell immune tolerance. Finally, GSEA showed that CDC42 expression was most significantly related to the Wnt signaling pathway. In conclusion, this study showed that CDC42 expression might be an important MDG in HCC and may prove to be a new gene for studying macrophage differentiation in HCC.

**Abbreviations**: HCC: hepatocellular carcinoma; TCGA: The Cancer Genome Atlas; ICGC: International Cancer Genome Consortium; GSEA: gene set enrichment analysis; GO: Gene Ontology; KEGG: Kyoto Encyclopedia of Genes and Genomes; ROC: receiver operating characteristic; K-M: Kaplan-Meier; AUC: the area under the ROC curve; TNM: Tumor size/lymph nodes/distance metastasis

## Graphical abstract

**Figure:**
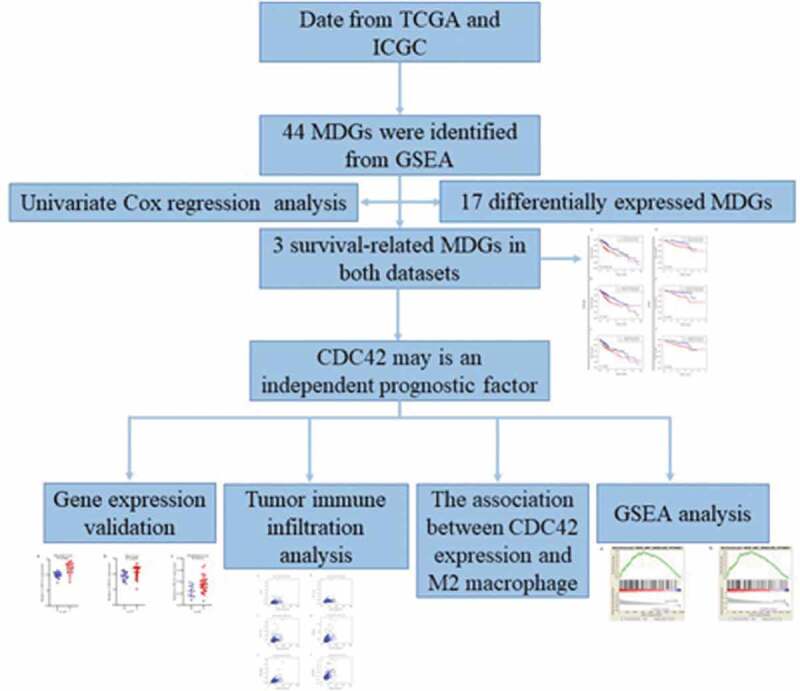

## Introduction

Hepatocellular carcinoma (HCC) ranks in the top five malignancies worldwide [1]. The incidence of HCC is increasing every year, and HCC has become the fastest-rising cause of cancer-related death [2]. Approximately 30% of newly diagnosed patients in the early stages are eligible for treatments by liver transplantation, hepatic resection, or percutaneous ablation [3] due to the invasion and metastasis of tumors. Thus, the elucidation of the underlying mechanisms involved in HCC hepatocarcinogenesis is of utmost importance.

Numerous studies have shown that tumor-associated macrophages (TMAs) favor tumor cells to modify the microenvironment and promote tumor growth, angiogenesis, invasion, and metastasis as well as suppress the antitumor immune response, including HCC [4]. Depending on their activators, macrophages are classified as either the classically activated (M1) subtype or the alternatively activated (M2) subtype [5]. M2 macrophages are associated with the development of an immunosuppressive microenvironment and enable the establishment and development of tumors as well as metastatic dissemination [5,6]. Recent studies on tumors have suggested that tumor-derived genes or lncRNAs mediate the regulation of macrophage polarization and activation [7–10]. For instance, HCC-derived exosomal lncRNA TUC339 modulates macrophage cytokine production, phagocytosis, and M1/M2 polarization [8]. Exosomes from metastatic osteosarcoma cells, by transferring TGFB2, promote the M2 phenotype and create immunosuppression. These findings substantiate the involvement of macrophages in HCC and suggest that tumor-derived MDGs may hold great importance for HCC development. Therefore, hub genes, involved in macrophage differentiation, are significant for improving patient prognosis and immunotherapy effectiveness.

Currently, the original mRNA microarray datasets and clinical prognostic information were downloaded from the ICGC and TCGA databases, which were used to identify prognosis-related MDGs. Then, biomarkers associated with HCC prognosis were elucidated by conducting a series of bioinformatics and survival analyses. Collectively, we identified that CDC42 might be a crucial gene in HCC carcinogenesis and macrophage differentiation.

## Materials & methods

### Data sources

The TCGA-LIHC RNAseq data and corresponding clinical follow-up information were downloaded from the publicly available TCGA database (https://tcga-data.nci.nih.gov/tcga/) and included 50 normal tissues and 374 primary HCC tissues. The public ICGC database (https://dcc.icgc.org/) contained 473 expression profiles and was selected for our analysis. The Oncomine database was used to compare the expression of CDC42 in HCC tissues with normal tissues (https://www.oncomine.org/) [11]. The CDC42 protein expression level and distribution in HCC tissues and liver tissues were analyzed using the Human Protein Atlas (HPA) database (https://www.proteinatlas.org/).

### Macrophage differentiation-associated gene (MDG) extract and differential expressed MDG analysis

A total of 44 genes were identified via the ‘GO_MACROPHAGE_DIFFERENTIATION’ gene set from the GSEA as MDGs (https://www.gsea-msigdb.org/gsea/msigdb/index.jsp). The R statistical software package ‘limma’ was applied to estimate differentially expressed MDGs. |log(FC)| ≥ 0.5 and adj. A P-value< 0.05 was used as the cutoff to identify differentially expressed MDGs [12]. Volcano plots and heat maps were generated using the ‘ggplot2’ and ‘pheatmap’ packages, respectively [13]. Both MDGs were identified using Venn diagrams.

### Survival analysis

To identify the prognostic value of the MDGs, Kaplan-Meier survival analysis was conducted from the TCGA and ICGC databases. MDG expression was further divided into high and low levels using the median expression level as the cutoff value. Our results were visualized using the ‘survival’ packages. Independent prognostic factors were evaluated by univariate Cox proportional hazards regression (UCR) analysis and multivariate Cox proportional hazards regression (MCR) analysis. P < 0.05 was considered statistically significant.

### Gene set enrichment analysis (GSEA)

GSEA has been extensively applied to identify underlying pathways [14]. According to the median expression of CDC42, samples were classified as high or low CDC42 levels. The C2.cp.kegg.v7.0.symbols.gmt dataset was obtained from the Molecular Signatures Database (MsigDB). NOM P-values <0.05, |NES| >1 and FDR q < 0.25 were considered statistically significant.

### Immune infiltration analysis

The TIMER database is a comprehensive resource for systematic analysis of immune infiltrates across diverse cancer types, which is validated using pathological estimations. The association between hub genes and tumor purity and immune checkpoint inhibitors (PD-1, PD-L1, and CTLA4) was also analyzed (https://cistrome.shinyapps.io/timer/) [15].

### Statistical analysis

All statistical analyses were conducted by RStudio software and SPSS v.23.0 software (IBM Corp.). Student’s *t*-test for independent samples was performed to assess the significant differences between the two groups. Spearman’s correlation analysis was used for the correlation analysis.

## Results

### Identification of differentially expressed MDGs

**Figure 1.**
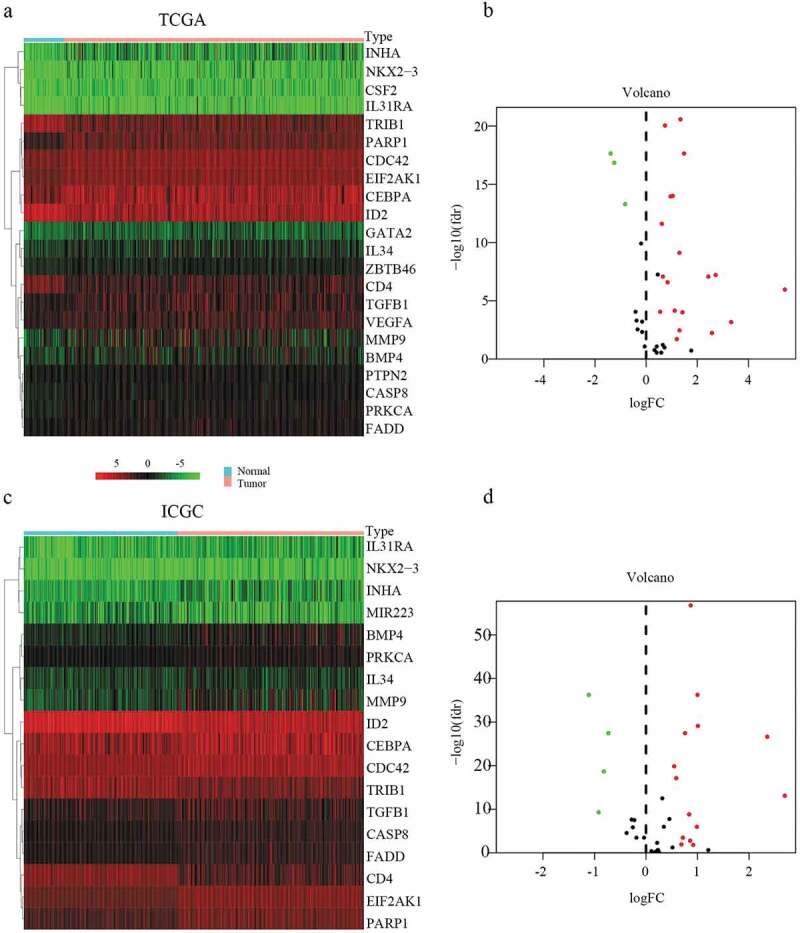
Heatmap and volcanic maps of differentially expressed MDGs from TCGA and ICGC data. (a and b) Differentially expressed MDGs obtained from the TCGA dataset. (c and d) Differentially expressed MDGs obtained from the ICGC dataset

**Figure 2.**
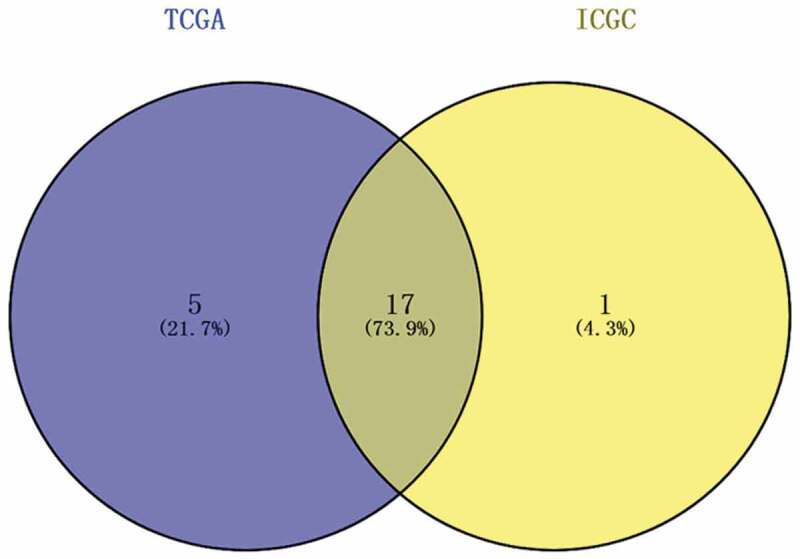
Venn diagram showing the intersecting differentially expressed MDGs from two public databases. Blue area: TCGA dataset; yellow area: ICGC dataset; cross area: differentially expressed MDGs expressed in both databases

**Figure 3.**
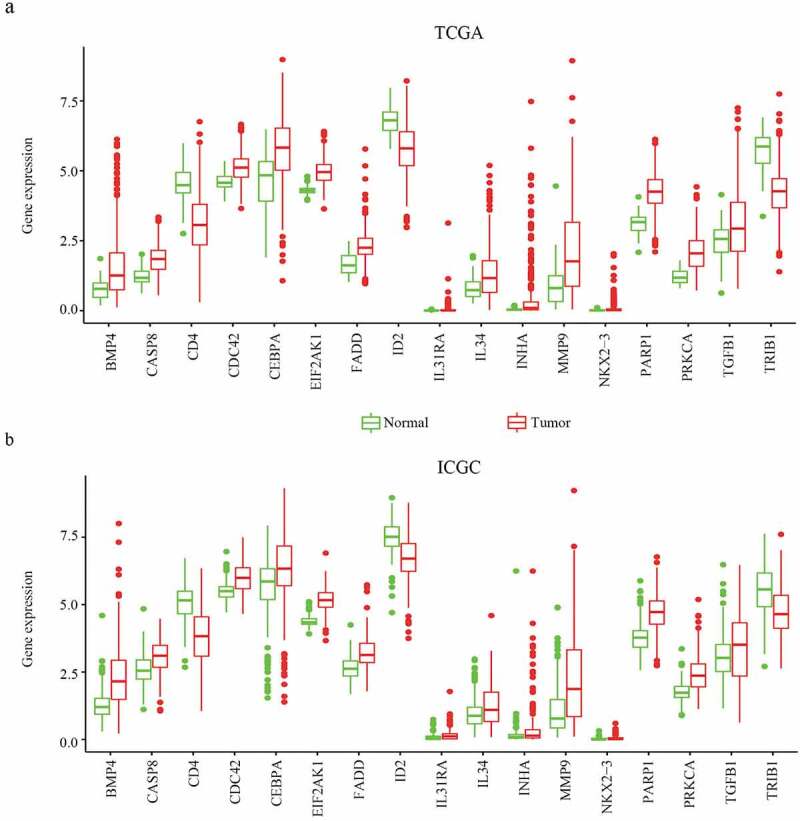
The expression of differentially expressed MDGs in HCC from TCGA and ICGC data. (a) The expression of differentially expressed MDGs in TCGA data. (b) The expression of differentially expressed MDGs in ICGC data

A total of 22 and 18 differentially expressed MDGs were identified from the TCGA and ICGC datasets, respectively (Figure 1). Moreover, Venn diagrams (Figure 2) revealed that 14 upregulated genes were commonly found in both tumor groups, as well as three downregulated genes (Figure 3 and Table 1).

**Table 1.** The specific information of DEGs

ID	Description Name	Expression
PARP1	Poly(ADP-ribose) polymerase 1	Down
INHA	Inhibin subunit alpha	Down
IL31RA	Interleukin 31 receptor A	Down
MMP9	Matrix metallopeptidase 9	Down
NKX2-3	NK2 homeobox 3	Down
BMP4	Bone morphogenetic protein 4	Down
PRKCA	Protein kinase C alpha	Down
IL34	Interleukin 34	Down
TGFB1	Transforming growth factor beta 1	Down
CEBPA	CCAAT enhancer binding protein alpha	Down
CASP8	Caspase 8	Down
FADD	Fas associated via death domain	Down
EIF2AK1	Heme-regulated eIF2α kinase	Down
CDC42	Cell division cycle 42	Down
ID2	Inhibitor of DNA binding 2	UP
CD4	CD4 molecule	UP
TRIB1	Tribbles pseudokinase 1	UP

### Identification of prognosis-related MDGs in the two datasets

**Figure 4.**
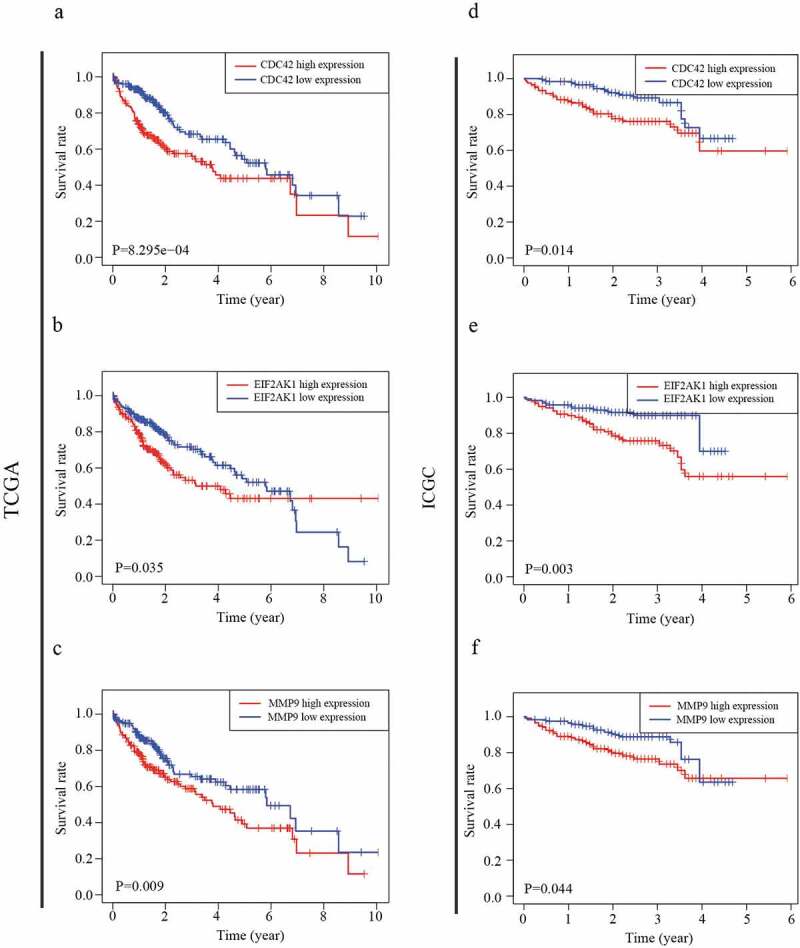
The relationship between MDG expression and the prognosis of HCC patients. (a–c) Kaplan-Meier estimated survival curves of CDC42, EIF2AK1 and MMP9 were constructed using TCGA clinical data. (d–f) Kaplan-Meier estimated survival curves of CDC42, EIF2AK1 and MMP9 were constructed using ICGC clinical data

**Figure 5.**
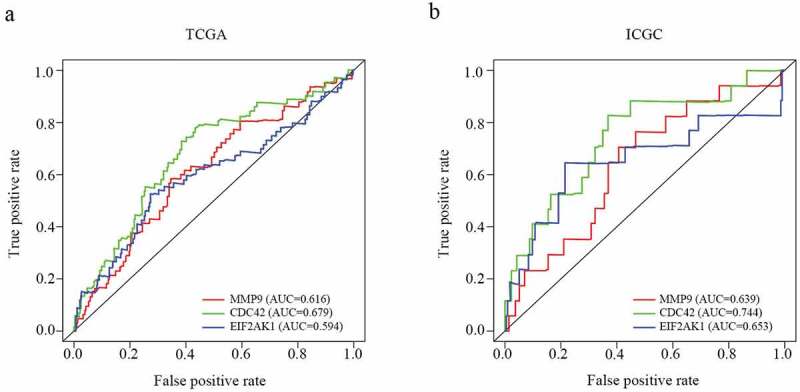
ROC analysis of the CDC42, EIF2AK1 and MMP9 signatures in HCC. (a) ROC analysis of the TCGA database. (b) ROC analysis of the ICGC database

Our results showed that three genes out of seventeen were significantly related to HCC patient prognosis in the two datasets. The patients who had a worse prognosis were those with a high expression of the three genes (CDC42, EIF2AK1 and MMP9) than those with a low expression of these genes (Figure 4(a–f)). To validate the predictive value of the three genes, an ROC curve was established (Figure 5(a,b)). The results indicated good predictive performance and repeatability of the group discrimination.

### CDC42, EIF2AK1 and MMP9 expression multivariate analysis

The independent predictive value of the CDC42, EIF2AK1 and MMP9 expression levels for OS were determined by UCR and MCR analyses with data from the TCGA and ICGC databases. In UCR analysis, CDC42 and EIF2AK1 expression levels were associated with overall survival (OS) in TCGA and ICGC databases (P *< *0.05) (Table 2). The MCR analysis showed that only the expression level of CDC42 could serve as an independent prognostic factor for HCC patients in both databases (P < 0.05) (Table 2), which indicated that CDC42 might be closely associated with HCC development.

**Table 2.** Univariate and Multivariate analyses of factors associated with survival of patients with HCC in TCGA database and ICGC database

	Overall survival(TCGA)					Overall survival(ICGC)			
	Univariate		Multivariate			Univariate		Multivariate	
Variable	pvalue		HR (95% CI)	pvalue		pvalue		HR (95% CI)	pvalue
Age, years (≥53 vs. <53)	0.591		1.011(0.991–1.031)	NA		0.800		0.314(0.157–0.628)	NS
Gender (male vs. female)	0.301		1.03(0.609–1.743)	NA		0.031		0.996(0.963–1.031)	NA
Grade (well or moderate vs. poor)	0.914		1.027(0.732–1.443)	NA		-		-	-
Stage(Ⅰ/ⅡVSⅠⅠⅠ/Ⅳ)	<0.001		1.181(0.425–3.278)	NA		<0.001		2.021(1.382–2.954)	<0.001
T(1/2 vs. 3/4)	<0.001		1.444(0.569–3.667)	NA		-		-	-
Distant metastasis (positive vs. negative)	0.023		1.752(0.456–6.735)	NA		-		-	-
Lymph nodes (positive vs. negative)	0.328		1.398(0.205–9.509)	NA		-		-	-
CDC42 expression (low vs. high)	<0.001		1.017(1.002–1.033)	0.030		0.001		1.016(1.004–1.027)	0.007
EIF2AK1 expression (low vs. high)	0.005		1.017(0.996–1.038)	NA		0.007		1.016(0.995–1.038)	NA
MMP9 expression (low vs. high)	0.034		1.003(0.998–1.007)	NA		0.960		0.991(0.975–1.007)	NA

NS, not significant; NA, not available .

### CDC42 is overexpressed in human HCC

**Figure 6.**
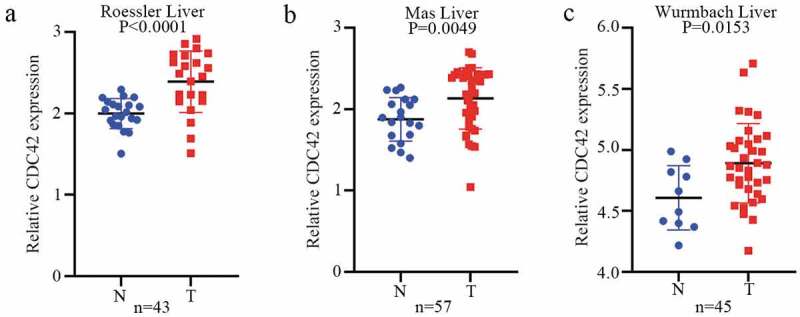
CDC42 expression is upregulated in primary HCC. (a) CDC42 expression in the Roessler liver dataset in Oncomine. (b) CDC42 expression in the Mas liver dataset in Oncomine. (c) CDC42 expression in the Wurmbach liver dataset in Oncomine

**Figure 7.**
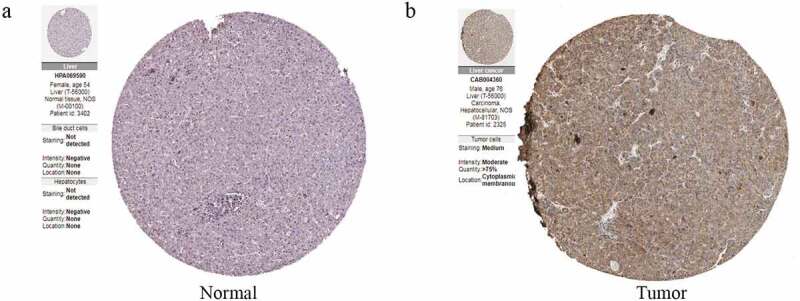
Immunohistochemistry confirmed the differential expression of CDC42. (a) The expression of CDC42 in normal tissue. (b) The expression of CDC42 in tumor tissue

To further determine the expression of CDC42 in HCC, we queried publicly available microarray datasets and checked CDC42 mRNA expression levels between HCC tissues and normal tissues using the Oncomine database. The results showed that the expression of CDC42 was much higher in cancer tissues than in normal tissues (P < 0.05) [16–18] (Figure 6 (a–c)). Immunohistochemistry for CDC42 showed that CDC42 was mainly expressed in the cytoplasm and cytoplasmic membrane but was almost undetectable in normal tissue (Figure 7 (a,b)). The results are consistent with the results from the TCGA and ICGC datasets.

### The correlation between CDC42 expression and immune cell infiltration

**Figure 8.**
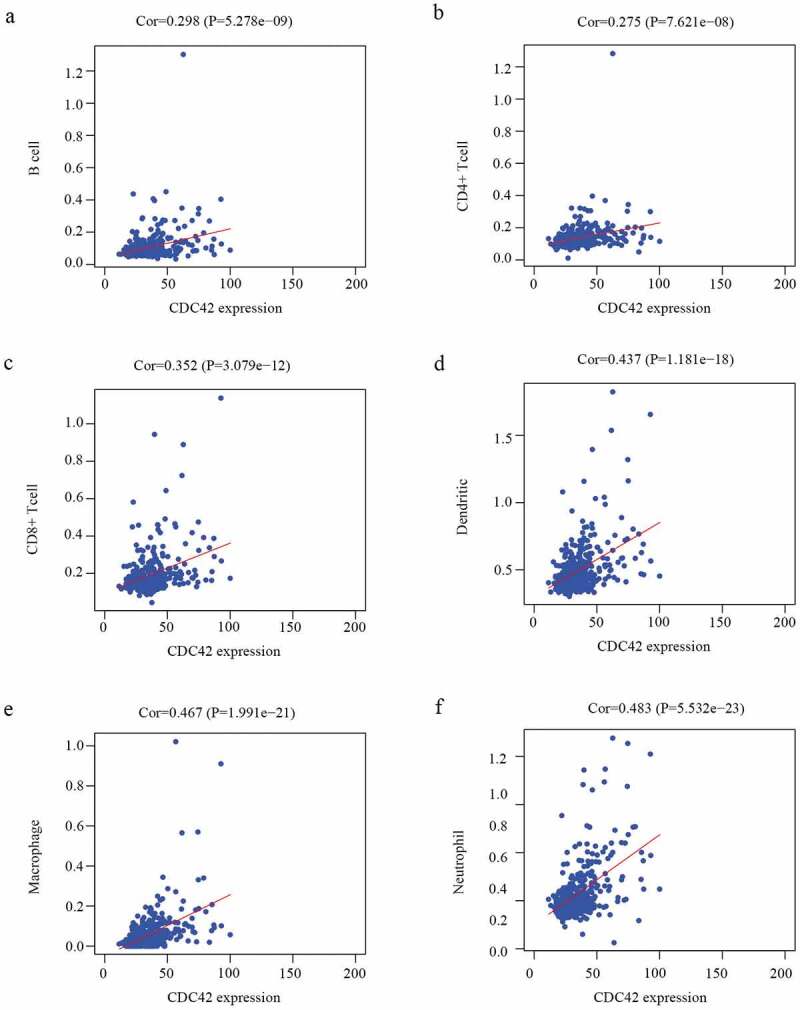
The immune infiltration levels of six immune cell types associated with CDC42 expression were analyzed based on the TIMER database. (a–f) CDC42 expression positively correlates with the infiltration of B cells (a), CD4 + T cells (b), CD8 + T cells (c), dendritic cells (d), macrophages (e), and neutrophils (f)

Immune cell infiltration was significantly associated with survival in diverse tumors. Therefore, further investigations were performed to evaluate the association between CDC42 expression and the immune infiltration characteristics of HCC patients. The results illuminated that CDC42 expression was positively related to B cells (r = 0.298 P = 5.278e-09), CD8 + T cells (r = 0.352 P = 3.079e-12), CD4 + T cells (r = 0.275 P = 7.62e-08), macrophages (r = 0.467 P = 1.991e-21), neutrophils (r = 0.483 P = 5.532e-23), and dendritic cells (r = 0.437 P = 1.181e−18) (Figure 8(a–f)). The results showed that CDC42 expression was closely related to tumor immunity. Therefore, the association between CDC42 expression and macrophages was further investigated.

### Expression levels of CDC42 are positively related to M2 macrophage markers

**Figure 9.**
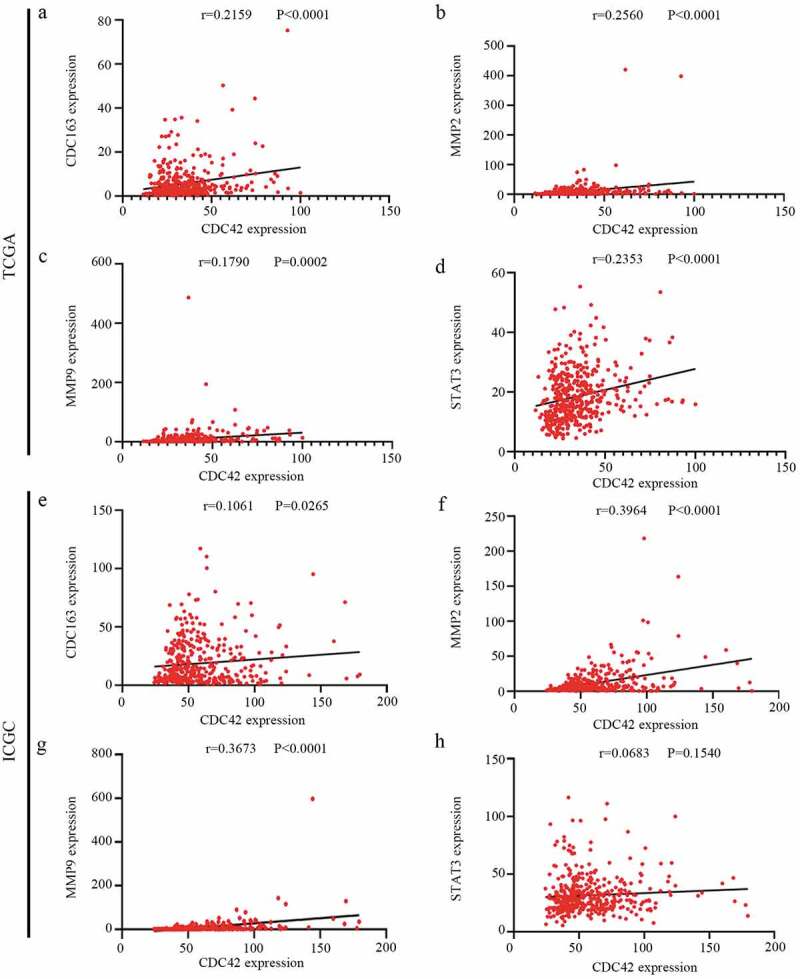
Correlation analyses between CDC42 expression and M2 macrophage markers. (a–d) The expression of CDC42 was significantly correlated with M2 macrophage markers (CD163, MMP2, MMP9, and STAT3) in the TCGA database. (e–h) The expression of CDC42 was significantly correlated with M2 macrophage markers (CD163, MMP2, MMP9, and STAT3) in the ICGC database

In our study, higher expression levels of CDC42, a macrophage differentiation-associated gene, were associated with worse prognosis. Previous studies have reported that higher infiltration of M2 macrophages promotes cancer occurrence, development and deterioration [5]. Therefore, we considered whether CDC42 expression was related to M2 macrophage differentiation. Then, the association between CDC42 expression and M2 macrophage markers (CD163, MMP2, MMP9, and STAT3) [4] was further investigated with data from the TCGA and ICGC databases. Interestingly, we found that CDC42 expression positively correlated with M2 macrophage marker gene expression in both databases (Figure 9(a–h)). These results might indicate that CDC42 expression may be associated with M2 macrophage differentiation. Previous studies have reported M2 macrophage polarization suppressed PD-1 immunotherapy [19], which indicated that CDC42 expression may be closely related to the tolerance of tumor immunotherapy. To verify this hypothesis, we analyzed the association between CDC42 expression and immune checkpoint inhibitors.

### Expression of CDC42 is positively related to immune checkpoint inhibitors

**Figure 10.**
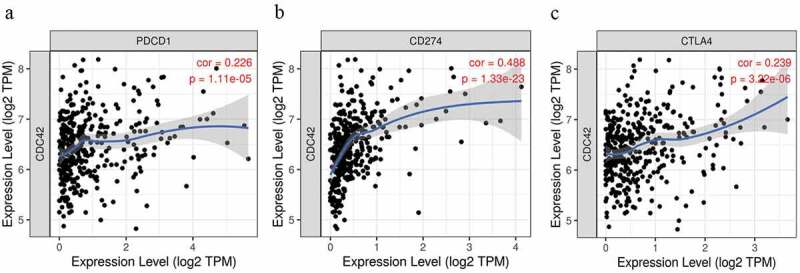
Correlation analyses between CDC42 expression and immune checkpoint inhibitors

Immune checkpoint inhibitor (PD-1, PD-L1, and CTLA4) expression levels are capable of accurately predicting the response to pembrolizumab or nivolumab [20,21]. In our results, the levels of CDC42 were positively associated with PD-1 (r = 0.226 P = 1.11e-05), PD-L1 (r = 0.488 P = 1.33e-23), and CTLA4 (r = 0.239 P = 3.22e-06) (Figure 10). The results showed that CDC42 expression may be related to the efficiency of immunotherapy.

(a–c) The expression of CDC42 was positively correlated with PD-1 (PDCD1), PD-L1 (CD274), and CTLA4.

### CDC42 positively associated with Wnt signaling pathway

**Figure 11.**
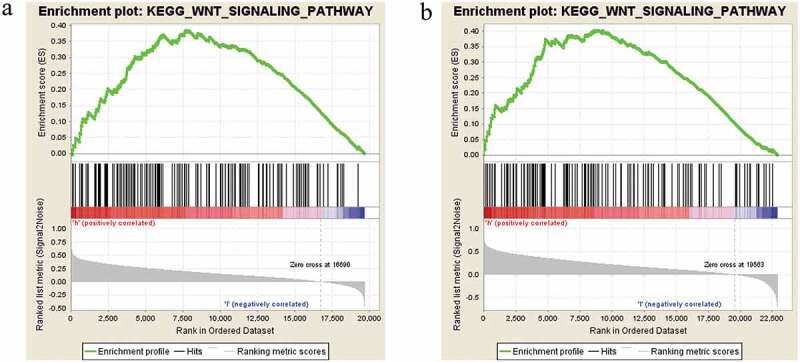
Enrichment plots from GSEA. (a) CDC42 expression positively correlated with the Wnt signaling pathway in the TCGA dataset. (b) CDC42 expression positively correlated with the Wnt signaling pathway in the ICGC dataset

To obtain further insight into the CDC42 expression involved in signaling pathways in HCC, GSEA was performed. GSEA showed that CDC42 expression was positively associated with the Wnt signaling pathway (Figure 11 (a,b)). Many studies have reported that the Wnt signaling pathway plays significant roles in regulating tumor macrophage differentiation, including HCC [22,23]. The GSEA results provide a significant molecular basis for better understanding how CDC42 plays a role in M2 macrophage differentiation.

## Discussion

Inflammation is considered to be an important factor in the development of a variety of malignancies. Tumor-associated macrophages (TAMs) are one of the most significant immune cells during chronic inflammation. TAMs are associated with colorectal liver metastasis [24], ovarian cancer [25], and HCC [26]. To identify MDGs associated with HCC development, we first used the TCGA and ICGC databases to identify seventeen differentially expressed MDGs. Second, the Kaplan–Meier curves demonstrated that the three MDGs were closely related to OS. UCR and MCR analyses confirmed that CDC42 expression can be used as an independent prognostic risk factor for HCC. Furthermore, CDC42 expression was positively related to six immune cell and M2 macrophage markers. Finally, GSEA indicated that CDC42 expression may affect the Wnt signaling pathways and promote M2 macrophage differentiation.

Cell division cycle 42 (CDC42), a member of the RHO GTPase family, regulates cytoskeletal reorganization in different ways [27]. Numerous studies have shown that CDC42 functions as a tumor promoter in various cancers and influences proliferation, motility, polarity, growth and drug resistance [28,29]. On the other hand, CDC42 is closely associated with immunity and inflammation. For instance, CDC42 mediates MTOC polarization in dendritic cells, which in turn influences the delivery of cytokines at immune synapses [30]. CDC42 controls the immune responses of iNKT cells by regulating the direction of IL-4 secretion [31]. Many studies have found that NK cells and T cells have the innate ability to kill HCC tumor cells in vitro [32,33]. In this research, CDC42 expression correlated with worse prognosis in HCC. There was a strong positive correlation with the infiltration of six immune infiltrates and the expression of three immune checkpoint inhibitors (PD-1, PD-L1, and CTLA4). Immunotherapy with immune checkpoint inhibitors is a promising approach for cancer treatment [34], and it has been used to treat various cancers, including lung, liver, and head and neck cancers [35–37]. Abnormal immune checkpoint marker expression is a risk factor for many diseases [38]. When these checkpoint molecules are overexpressed, immune functionality may be suppressed [39]. This indicated that CDC42 expression levels may be associated with tumor immunity and immunotherapy.

Our results showed that CDC42 expression was positively associated with the Wnt signaling pathway. The Wnt signaling pathway, a key cell proliferation and differentiation pathway, has been associated with dysregulated macrophage activity in disease [40–42]. For instance, the activation of Wnt/β-catenin signaling leads to the inhibition of M1 macrophage polarization but the promotion of M2 polarization [42]. Wnt5a enhances transforming growth factor β1 (TGF-β1)-induced macrophage M2 polarization in kidney fibrosis [43]. Researchers have previously reported that CDC42 promotes Schwann cell proliferation and migration through the Wnt/β-Catenin signaling pathway after sciatic nerve injury [44]. CDC42 and noncanonical Wnt signal transduction pathways cooperate to promote cell polarity [45]. This indicated that CDC42 may associate with the Wnt/β-Catenin signaling pathway. However, the relationship between CDC42 and the Wnt/β-Catenin signaling pathway in HCC tumorigenesis and M2 macrophage differentiation needs to be further elucidated.

## Conclusions

In summary, we identified that CDC42 expression may be an independent prognostic factor in HCC patients via UCR and MCR analyses with data from the TCGA and ICGC datasets, and we found that CDC42 may affect the Wnt signaling pathway and M2 macrophage differentiation, which may prove to be a new gene for studying macrophage differentiation in HCC. However, these results need to be further validated in future studies.

## Supplementary Material

Supplemental Material

## Data Availability

The datasets analyzed was acquired from The Cancer Genome Atlas (TCGA) database (https://portal.gdc.cancer.gov/) and ICGC database (https://dcc.icgc.org/).

## Highlights

(1) CDC42 expression may be an independent prognostic factor in HCC patients.

(2) CDC42 expression was positively related to immune cell infiltration and M2 macrophage markers.

(3) CDC42 expression was positively associated with the Wnt signaling pathway.
