# Supplementary figures and images for: Identification of hepatocellular carcinoma-related genes associated with macrophage differentiation based on bioinformatics analyses
Source: Bioengineered. 2021 Jan 18;12(1):296–309. doi: 10.1080/21655979.2020.1868119 (PMC8806327; doi:10.1080/21655979.2020.1868119)

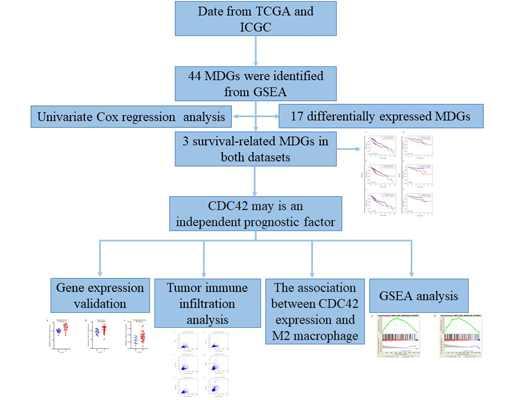

Supplement: Supplemental Material [file KBIE_A_1868119_SM8751.tif]
